# Application of the Ilizarov technique for knee joint arthrodesis as a treatment for end-stage tuberculosis of the knee

**DOI:** 10.1186/s12891-020-03603-9

**Published:** 2020-08-26

**Authors:** Jiachen Sun, Qiang Li, Feng Gao, Zhou Xiang, Qi Huang, Lang Li

**Affiliations:** 1 Department of Orthopedics, West China Hospital, Sichuan University, Guoxue Lane 37, Chengdu, 610041 Sichuan Province People’s Republic of China; 2 Department of Orthopaedics, Hospital of Chengdu Office of People’s Government of Tibetan Autonomous Region, NO.20 Ximianqiao Cross Street, Chengdu, 610041 Sichuan People’s Republic of China

**Keywords:** Ilizarov technique, End-stage tuberculosis of the knee, Arthrodesis, Anti-tuberculosis drugs

## Abstract

**Background:**

With the global determination to eliminate tuberculosis (TB), the treatment for end-stage TB of the knee joint is still a great clinical challenge. This study aims to retrospectively determine the clinical and radiographic outcomes after use of the Ilizarov technique for knee joint arthrodesis as a treatment for end-stage knee TB.

**Methods:**

Twenty-six patients with end-stage knee TB treated by external fixation with the Ilizarov fixator between 2012 and 2017 were examined. Anti-TB drugs were administered preoperatively, intraoperatively, and postoperatively. Clinical and radiologic examinations were performed for comprehensive evaluations, and these include C-reactive protein (CRP), erythrocyte sedimentation rate (ESR), flexion and valgus angle of the knee, leg-length discrepancy, and Lysholm score.

**Results:**

Twenty-four patients were followed up for an average of 5.8 years (2.2–7 years). All patients achieved bone fusion within a mean of 6.4 months (4–16 months). The ESR and CRP concentrations were observed to return to normal within 5.1 ± 1.1 months postoperatively. There was no recurrence of TB. At last follow-up, the mean leg-length discrepancy was 2.7 ± 1.4 cm, and the mean alignment was 8.7 ± 2.6° flexion and 5.3 ± 1.0° valgus. No patient had a significant rotational deformity. The average Lysholm score was seen to improve significantly from 36.8 ± 18.4 preoperatively to 79.5 ± 5.9 at final follow-up (*p* < 0.0001).

**Conclusion:**

This study has demonstrated that the Ilizarov technique for knee joint arthrodesis as a treatment of end-stage knee TB achieved promising outcomes with minimal complications.

## Background

Although the annual incidence rate of tuberculosis (TB) is seen to be gradually decreasing, about 25% of the world’s population is still at risk of developing it, with more than 1 million deaths annually [1, 2]. The goal to end the global TB epidemic by 2030 was committed to by all the United Nations Member States; however, because of the economic, literacy, medical, and transportation limitations in some areas, realizing this goal still requires international efforts. Except for pulmonary TB, morbidity from TB is the highest in the skeletal system, for which knee joint TB ranks second only to that of the spine (50%) and close to that of the hip joint (10%) [3, 4]. Early synovial TB or bone TB of the knee joint can be treated by anti-TB drugs, joint cavity cleaning, and thorough debridement; these are often associated with good outcomes [5, 6]. However, end-stage total joint TB is frequently accompanied by severe destruction of the articular cartilage, joint deformities, and severe dysfunction, which often require open debridement and arthrodesis to relieve inflammation and pain and to maintain a near-normal gait [6, 7].

Because of the difficulty of early diagnosis and the relatively low level of economic and transportation resources in some areas, many patients with TB of the knee are often referred for medical treatment late in the disease course or when it is already terminal [8]. For this condition, arthrodesis has been identified as the main treatment option. Currently, several techniques have been considered for arthrodesis, including internal fixation with plates and screws, intramedullary nailing, and external fixation with a unilateral, bilateral, or circular external fixator [9, 10]. However, none of these approaches has been proven to be the best choice for the treatment of end-stage TB of the knee as an arthrodesis technique. Meanwhile, the Ilizarov technique, characterized by the tension–stress principle, has been widely used in orthopedic treatment of lower limb fractures, bone defects, and ankle diseases [11–13]. This approach can be used to treat knee joints with total knee arthroplasty failure, osteoarthritis, or septic knee as a method for joint fusion [14–16]. To date, this technique for the treatment of end-stage TB of the knee has only been reported in a case report, without detailed surgical procedure and long-term follow-up [17].

In terms of the number of TB cases, China ranks the second highest in the world (9%) [1]. In Western China, where there are many patients reported to be suffering from end-stage knee TB, economic and medical resources are relatively weak [18]. Therefore, what these patients need is an appropriate arthrodesis technique with low cost, no need for secondary operation, and good curative effect. This retrospective study evaluates the efficacy of Ilizarov technique for the treatment of end-stage TB of the knee, and it is the hope of these authors to provide support for patients in need of this treatment and for their physicians.

## Methods

This study was approved by the Human and Ethics Committee for Medical Research at Hospital of Chengdu Office of People’s Government of Tibetan Autonomous Region, in accordance with the Declaration of Helsinki. Written informed consent was obtained for all patients and the parents or legal guardians of the children prior to the inclusion in the study.

Only patients who underwent single-stage arthrodesis of the knee using Ilizarov annulus external fixation for the treatment of end-stage knee TB were included in the study, and those with a follow-up period less than 2 years were excluded. Twenty-six patients met the inclusion criteria and underwent unilateral knee fusion using Ilizarov fixation (Boxia, China), and were evaluated retrospectively from October 2012 to October 2017 (Table 1). Four patients were not included because the follow-up time was less than 2 years. Of these 26 patients included, 18 were male and 8 female, with a mean age of 41.3 years (17–61 years), and cases comprised 18 in the left knee and 8 in the right knee. The mean duration of illness was 3.3 years (11 months–10 years). Before operation, a preliminary diagnosis was made based on the patient’s medical history, clinical symptoms, laboratory data, and radiographic examinations. All patients had low-grade fever, knee swelling and pain, and flexion deformities of the knee joint in varying degrees, including periarticular abscess of the leg in five cases and formation of a sinus cavity in and around the knee joint in eight cases. Most of them walked with pain and had abnormal gait, and a few patients need the assistance of crutches or wheelchairs. The erythrocyte sedimentation rate (ESR) and C-reactive protein (CRP) concentrations for all patients were higher than normal, and our findings revealed that these patients had not previously received continuous standard anti-TB treatment. They were positive for some of the classical laboratory examinations, including the synovial fluid polymerase chain reaction (PCR) testing, the DNA sequencing of TB bacillus, the purified protein derivative test, and the blood TB antibody test. Of these 26 patients, 2 patients had arthroscopic synovectomy and 1 underwent surgical debridement arthrotomy 10 years ago, and they were diagnosed with knee tuberculosis by pathologic analysis of biopsy specimens. Radiographic examination showed that all the patients had varying degrees of osteoporosis, uneven joint space narrowing, joint bone destruction, and sequestration. Bone destruction, location of dead bone, and cold abscess were observed by computed tomography (CT). Magnetic resonance imaging (MRI) showed synovial hypertrophy, an unclear boundary of surrounding soft tissue, and a clear scope of cold abscess.

**Table 1:** Patient characteristics

Characteristics (***n*** = 26)	Value
Age, years, mean ± SD	41.3 ± 9.6
Sex, M/F	18/8
Nationality, Tibetan/Han	19/7
Side, L/R	18/8
Duration, years, mean ± SD	3.3 ± 2.4
Pre-operation ESR, mm/h, mean ± SD	49.1 ± 27.5
Pre-operation CRP, mg/L, mean ± SD	41.2 ± 37.9
Pre-operation Lysholm score, mean ± SD	36.8 ± 18.4
Previous surgery, arthroscopic synovectomy/ debridement arthrotomy	2/1

*ESR* erythrocyte sedimentation rate, *CRP* C reactive protein

### Preoperative evaluation

**Fig. 1:**
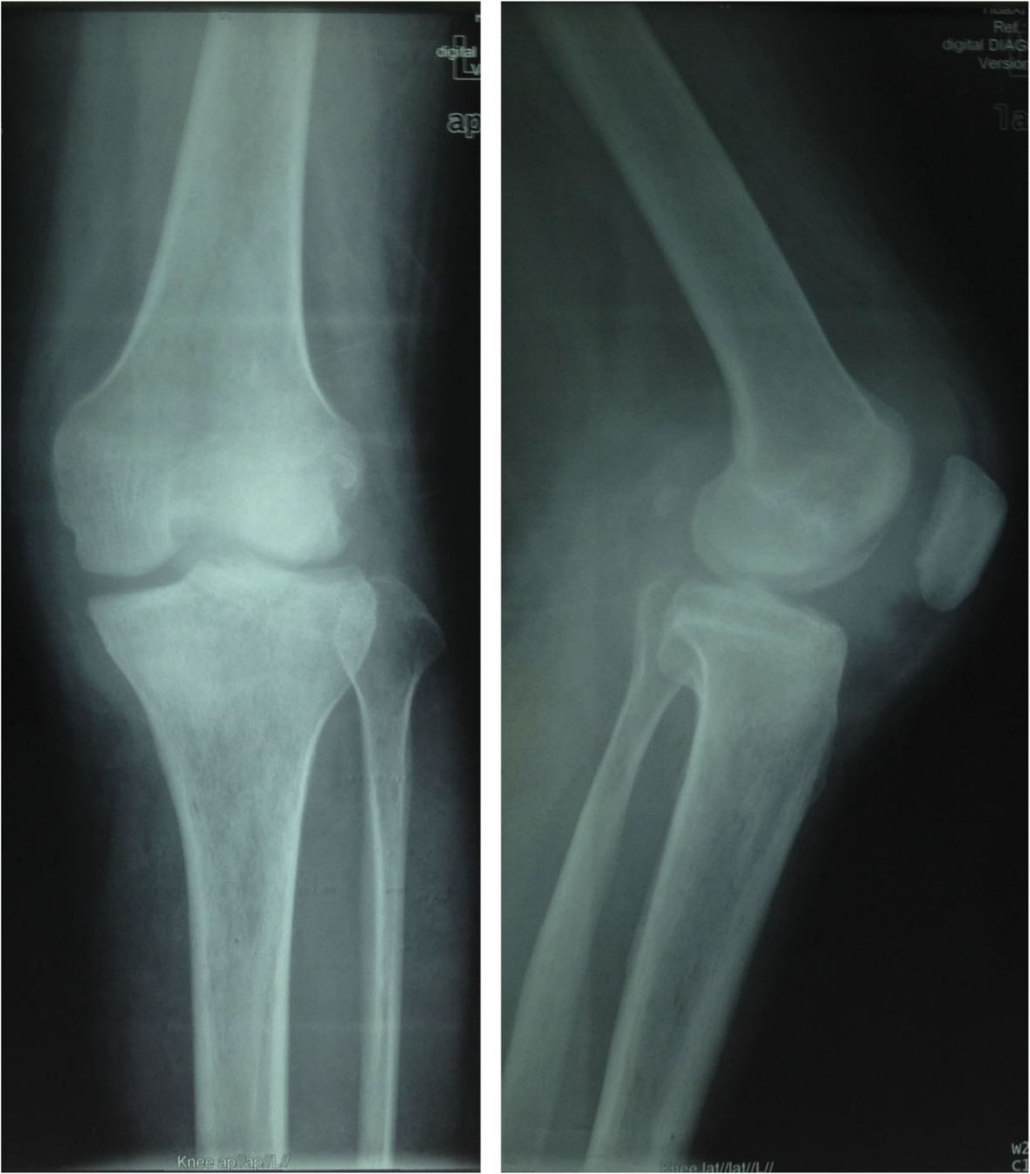
X-ray (anteroposterior and lateral): clearly osteoporosis, uneven joint space narrowing, and joint bone destruction

**Fig. 2:**
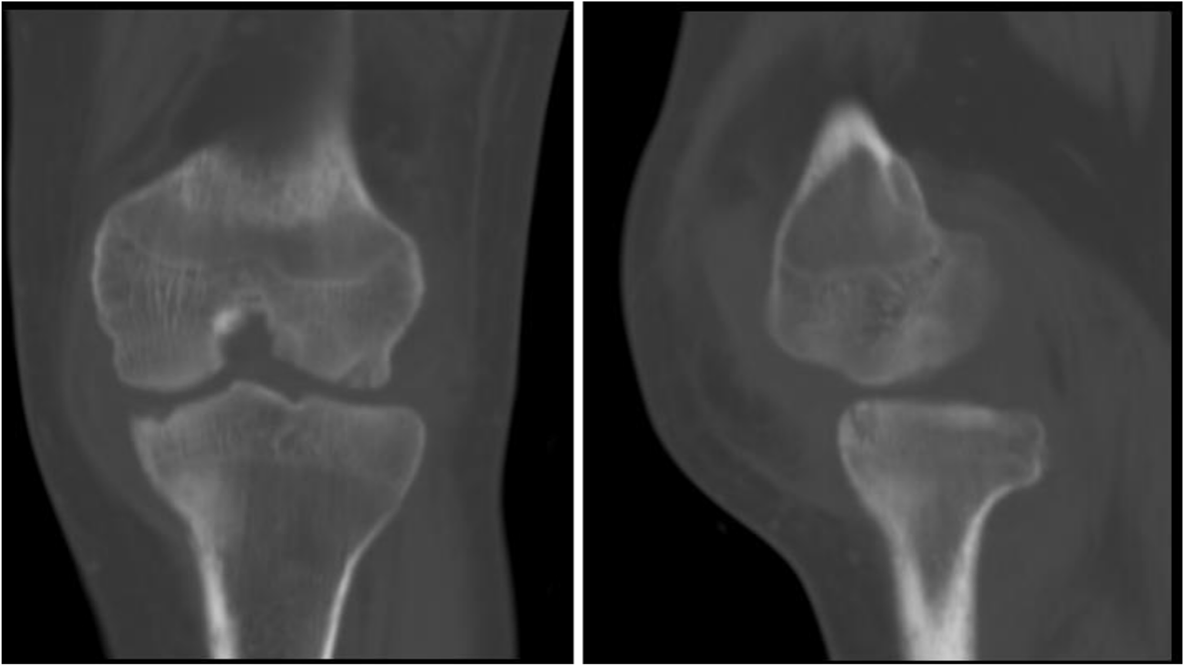
CT (anteroposterior and lateral): severe joint destruction and central and peripheral erosions

**Fig. 3:**
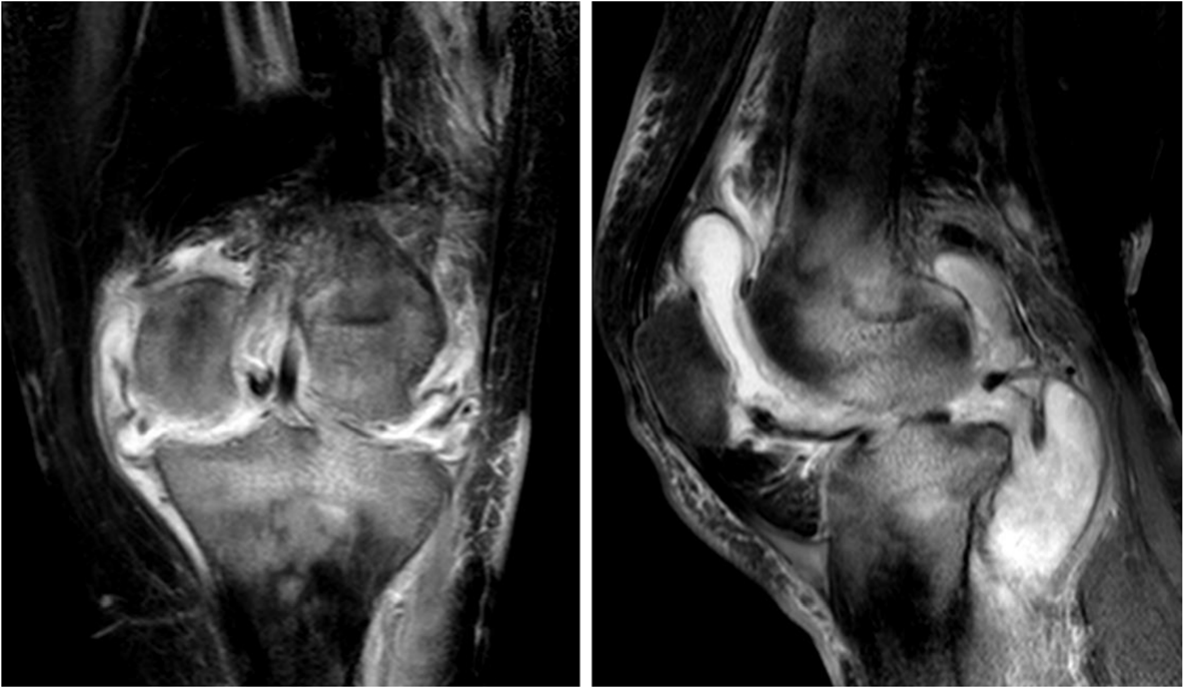
MRI (anteroposterior and lateral): synovial hypertrophy, unclear boundary of surrounding soft tissue, clear scope of cold abscess, severe joint destruction, obscure subcartilaginous bone plates, and central and peripheral erosions

All patients were confirmed to either have current or previous TB infection using the Mantoux test and chest radiography before surgery. After clinical, laboratory, and imaging examinations, the patients were diagnosed with end-stage knee TB, with the symptoms of total joint erosion, severe bone destruction, disappearance of joint space, and joint deformity and dysfunction. In addition, some patients had gravitation abscess and secretions from the sinus tract. The patients’ diets were then regulated, increasing high-protein food intake, and afterwards, the nutritional status of patients was seen to improve. All patients were treated with oral drugs (rifampin 450 mg/day, isoniazid 300 mg/day, and ethambutol 750 mg/day) and intramuscular streptomycin (800 mg/day, replacing streptomycin with levofloxacin in cases of allergy) against TB for 2–4 weeks. The patients with mixed infections received sensitive antibiotic treatments until the infection was controlled. Surgery was scheduled for patients if the following criteria are met: (1) when ESR and CRP concentrations showed a continuous downward weekly trend, (2) anemia and hypoproteinemia returned to normal, and (3) the pain and swelling of the knee were relieved. The diameters of the thigh and calf were measured preoperatively. Based on these measurements, the Ilizarov steel ring and Kirschner wire were selected, followed by sterilization. Preoperative examinations by radiography (RADspeed, Shimadzu, Japan), CT (Aquilion 16, Toshiba, Japan), and MRI (Achieva 3.0 T, Philips, the Netherlands) of the diseased knee were then performed (Figs. 1, 2 and 3). The extent of gravitation abscess was determined by MRI. Isoniazid and levofloxacin injection and streptomycin powder were also prepared for the operation.

### Surgical technique

**Fig. 4:**
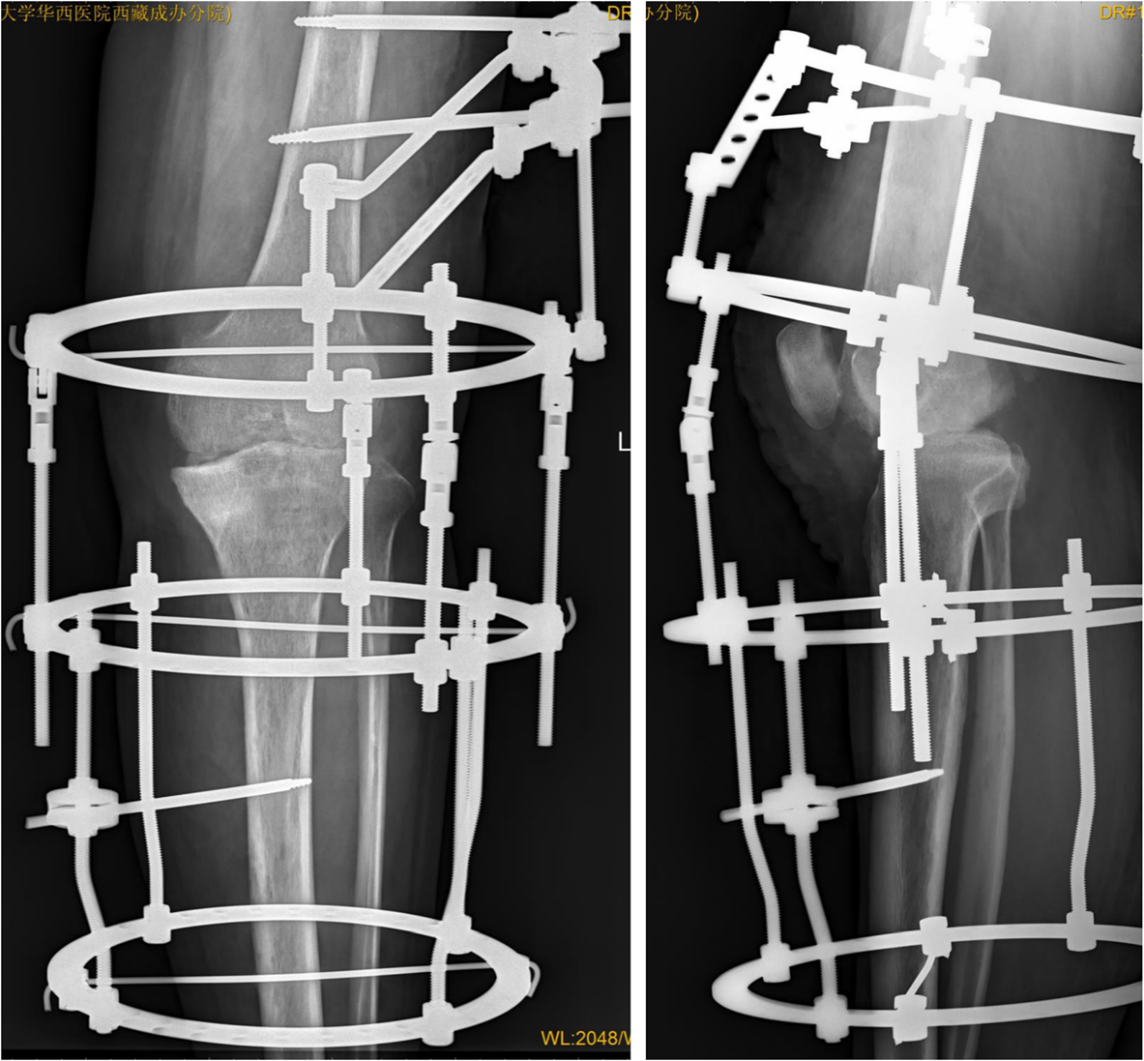
X-ray (anteroposterior and lateral): good bony contact after operation

**Fig. 5:**
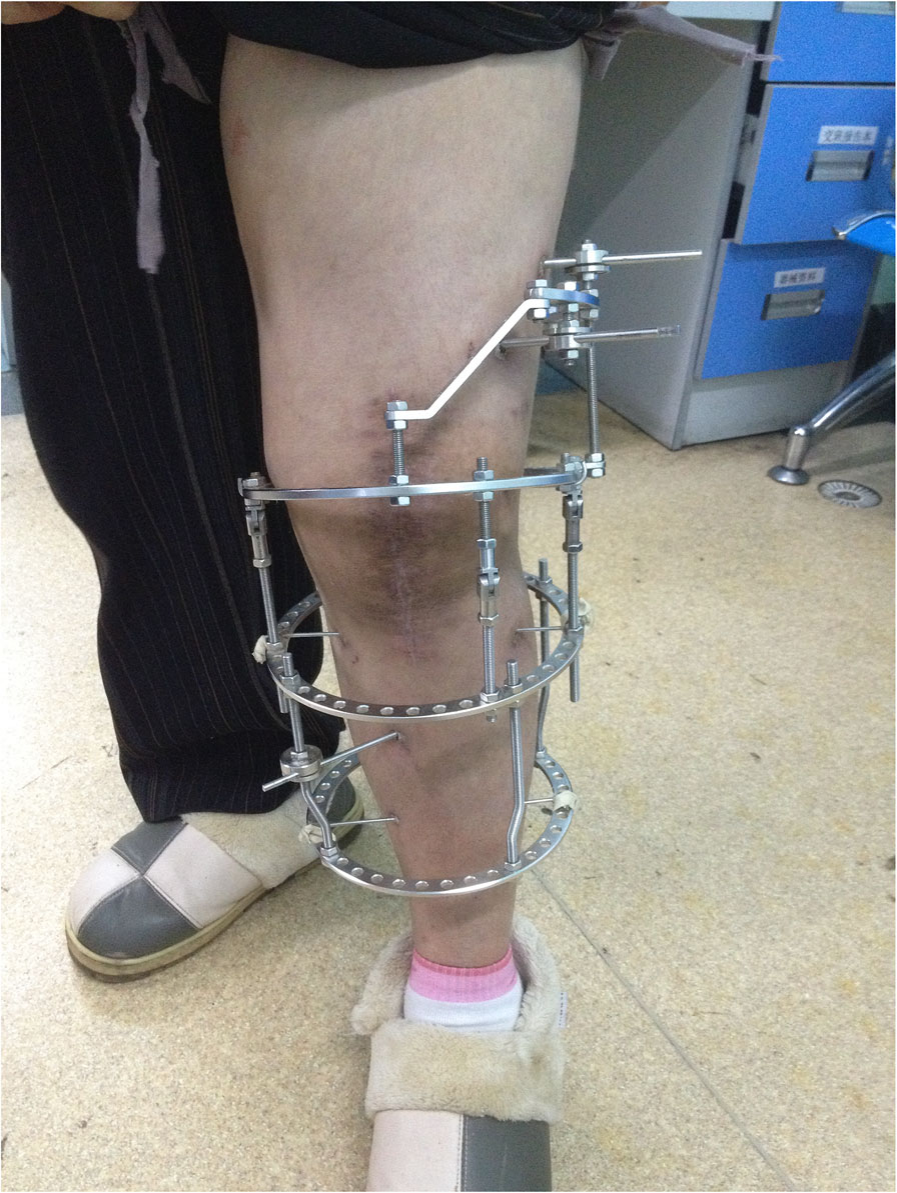
Clinical photograph of the patient after operation

All patients were placed in the supine position, administered inhalation and femoral nerve block anesthesia, and had a pneumatic tourniquet placed at the most proximal point of the affected limb (pressure of 260–300 mmHg based on the patient’s thigh diameter). The operation was divided into three steps. First, thorough debridement of the knee joint was performed. Either a midline incision was created or a previous incision was used, followed by an incision made in the joint capsule at the red and white line in the quadriceps tendon junction. The patella was then everted and preserved in preparation for two-stage joint replacement surgery if possible. Further extending the incision was considered based on the location of the gravitation abscess shown on MRI. Thorough debridement was realized through clearing all the sequestrum, granulation, pus, and cheese-like tissue until a fresh bone surface was evident. The bone surface was properly trimmed to make the interface between the femur and tibia more consistent. Samples of granulation tissue and bone were collected for pathologic and microbiologic studies. The incision site was then soaked with hydrogen peroxide twice (for 3 min at each application) and povidone for 10 min and then rinsed with 3 l of saline. Two doses of isoniazid and levofloxacin by injection and streptomycin powder were evenly administered into the operative site. The tourniquet was released after the site was bandaged. Second, the Ilizarov external fixation was applied. The steel rings were assembled at the beginning of the operation, and the lower limb was placed into them, including a half ring at the middle segment of the thigh, a full ring near the knee joint, and two full rings at the upper and middle part of the calf. Kirschner wires with a diameter of 2.5 mm and Schanz screws were used to attach the fixator, with the bolt unlocked during this time. Finally, the force line of the knee joint was adjusted, and then the fixator was locked. Both the tibial and femoral condyles were aligned to the maximum extent, and the femur and tibia were placed in 5° valgus and 5–10° flexion. Once the optimal position of the fusion was confirmed by fluoroscopy, the bolts of external fixator were locked (Figs. 4 and 5). The operative site was sterilized and sutured followed by bandaging under pressure.

### Postoperative management and assessment

The final diagnosis was confirmed by postoperative pathologic and microbiologic examinations. Langhans giant cells, epithelioid cells, and acid-resistant rod-shaped bacteria were found in all samples, and the PCR results of all samples were positive. The oral drugs of the preoperative anti-TB regimen were continued for 18 months after surgery except for intramuscular streptomycin (800 mg/day, which was continued only for 3 months, using levofloxacin instead in cases of allergy) or until the serum levels of inflammatory markers became normal (testing was continued at least 3 times per week with 2-week intervals), accompanied by strengthening of nutritional support, blood transfusion, and other treatments. Patients were taught to squeeze and fix the skin with gauze around the fixed needle, rinse the pin insertion sites with saline, and smear the pin sites with mupirocin ointment (Bactroban, Tianjin Smith Kline & French Laboratorles Ltd., China). Stitches were removed from the incision 3 weeks postoperatively. On postoperative day 1, patients could mobilize by non-weight-bearing movement with double crutches, and targeted exercise of iliopsoas and quadriceps femoris was recommended. On postoperative day 4, patients could walk with partial weight-bearing after observing no incision-site bleeding. The partial weight-bearing walking started from 5 kg and was increased by 5 kg every other day. Patients could begin walking with full weight from 3 to 4 weeks postoperatively, and crutches could be completely discarded at 8 weeks after surgery.

All patients were assessed comprehensively using both clinical and radiographic examinations. Blood samples were collected at each visit for the routine analysis of liver and renal function, CRP, ESR, and white blood cell counts. Radiographic examinations with anteroposterior and lateral views of the knee joint were performed every month, and the time of external fixator removal was determined according to the condition of bone fusion. The flexion angle, valgus angle, and the leg-length discrepancy were detected in the last follow-up using radiographic examination as just described and an additional full-length radiograph. The function of the knee joint, pain, and self-reported observations of patients pre- and postoperatively were evaluated using the Lysholm score. A score of 95–100 was considered excellent, 84–94 good, 65–83 fair, and less than 65 poor [19]. Clinical union was defined as the ability to walk without pain or tenderness at the arthrodesis site, and radiographic union was defined as the appearance of a circumferential bridging callus on both the anteroposterior and lateral films, which was confirmed by three experienced orthopedic surgeons [20].

### Statistical analysis

Statistical analyses were conducted with SPSS Version 16.0 software (SPSS Inc., Chicago, IL, USA). All measurement data were tested for normal distribution using the Kolmogorov-Smirnov Z test. Comparisons of variables between baseline and the endpoint were analyzed using paired t-tests when the distribution was normal; otherwise, the Wilcoxon signed-rank test was used. A *P* value of less than 0.05 was defined as significant, and it was considered highly significant when it was less than 0.01.

## Results

The results of the treatment and follow-up are shown in Table 2. Two patients were lost to follow-up after hospital discharge, and the mean follow-up time for the remaining 24 patients was 5.8 years (2.2–7 years). Among all 26 patients, 1 developed superficial wound infection, which healed after dressing change and antibiotics treatment, and 2 developed delayed wound healing. Among the 24 follow-up cases, 2 patients developed pin tract infection with local skin redness, swelling, and exudation within 1 month postoperatively, which were relieved after wound care and oral antibiotics; meanwhile, 1 patient developed pin tract pain at 3 months postoperatively, which was relieved after reducing exercise. No cases of external fixator failure have occurred. Bone fusion was observed on radiography in all follow-up patients, and the mean time to fusion was determined to be at 6.4 months (4–16 months). Delayed bone union occurred in only 1 patient, for whom full fusion was obtained at 16 months postoperatively after a prolonged external fixator time. One patient underwent total knee arthroplasty to obtain joint function 5 years after arthrodesis.

**Table 2:** Results of treatment and follow-up

Results (***n*** = 24)	Value
Total number of cases with complications	4
Cases with pin tract infection	2
Cases with pin tract pain	1
Cases with delayed bone union	1
Time for ESR and CRP to return to normal, months, mean ± SD	5.1 ± 1.1
Time to fusion, months, mean ± SD	6.4 ± 2.0
Post-operation Lysholm score, mean ± SD	79.5 ± 5.9
Leg-length discrepancy, cm, mean ± SD	2.7 ± 1.4
Post-operative knee joint flexion, °, mean ± SD	8.7 ± 2.6
Post-operative knee joint valgus, °, mean ± SD	5.3 ± 1.0

*ESR* erythrocyte sedimentation rate, *CRP* C reactive protein

**Fig. 6:**
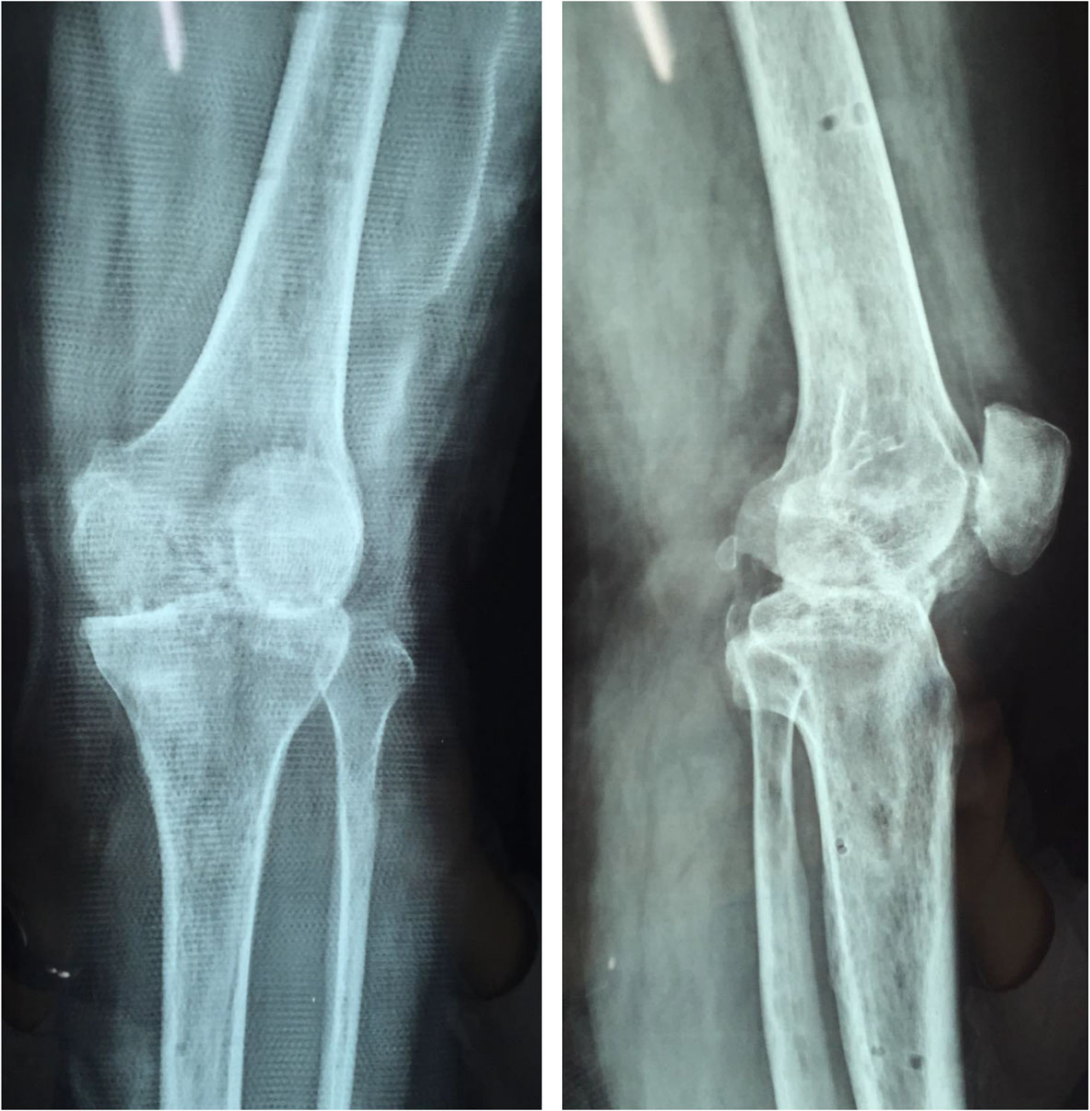
X-ray (anteroposterior and lateral): good knee fusion after removal of the external fixator (8.5 months after surgery)

**Fig. 7:**
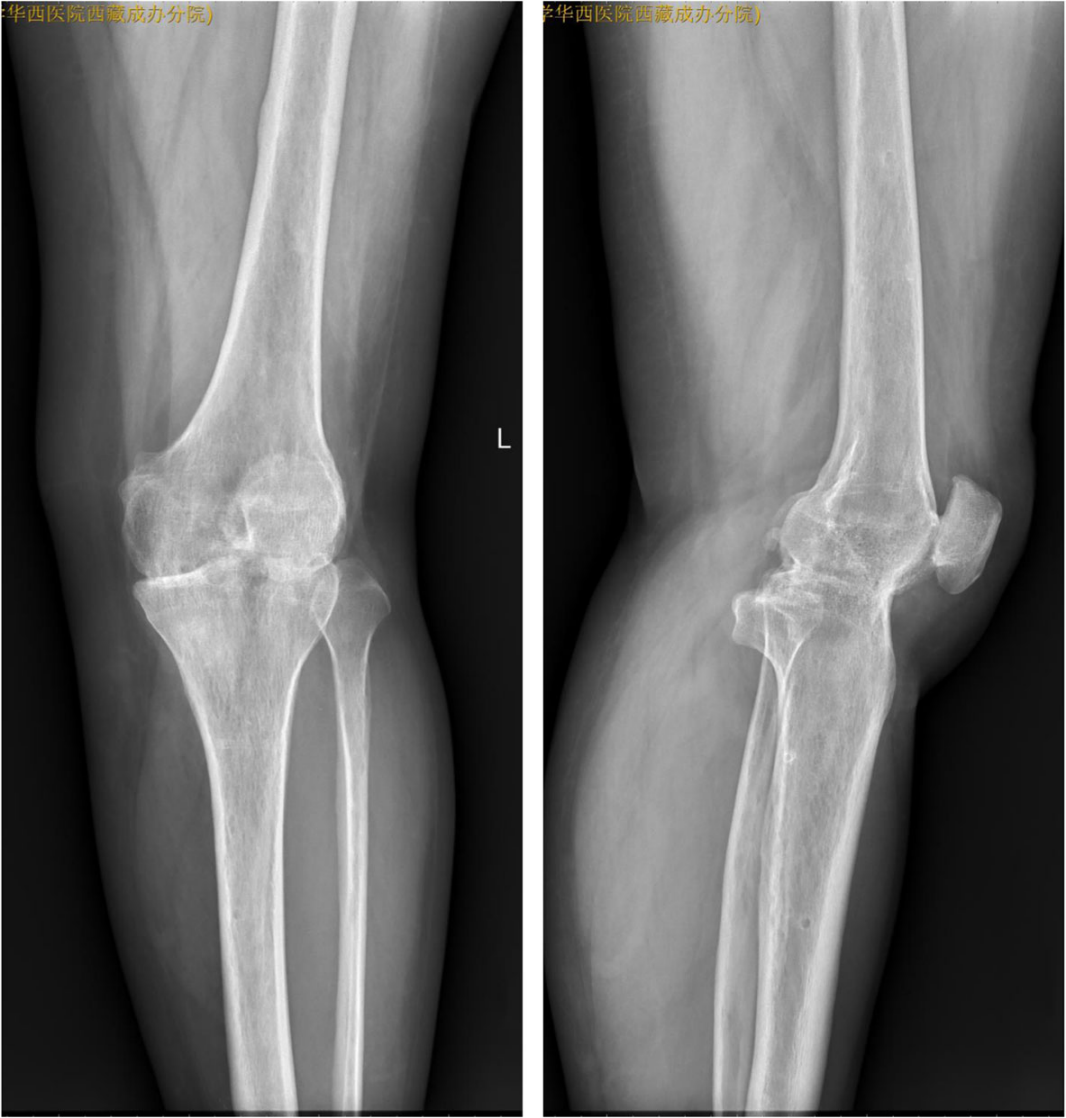
X-ray (anteroposterior and lateral): successful knee joint fusion (26 months after operation)

**Fig. 8:**
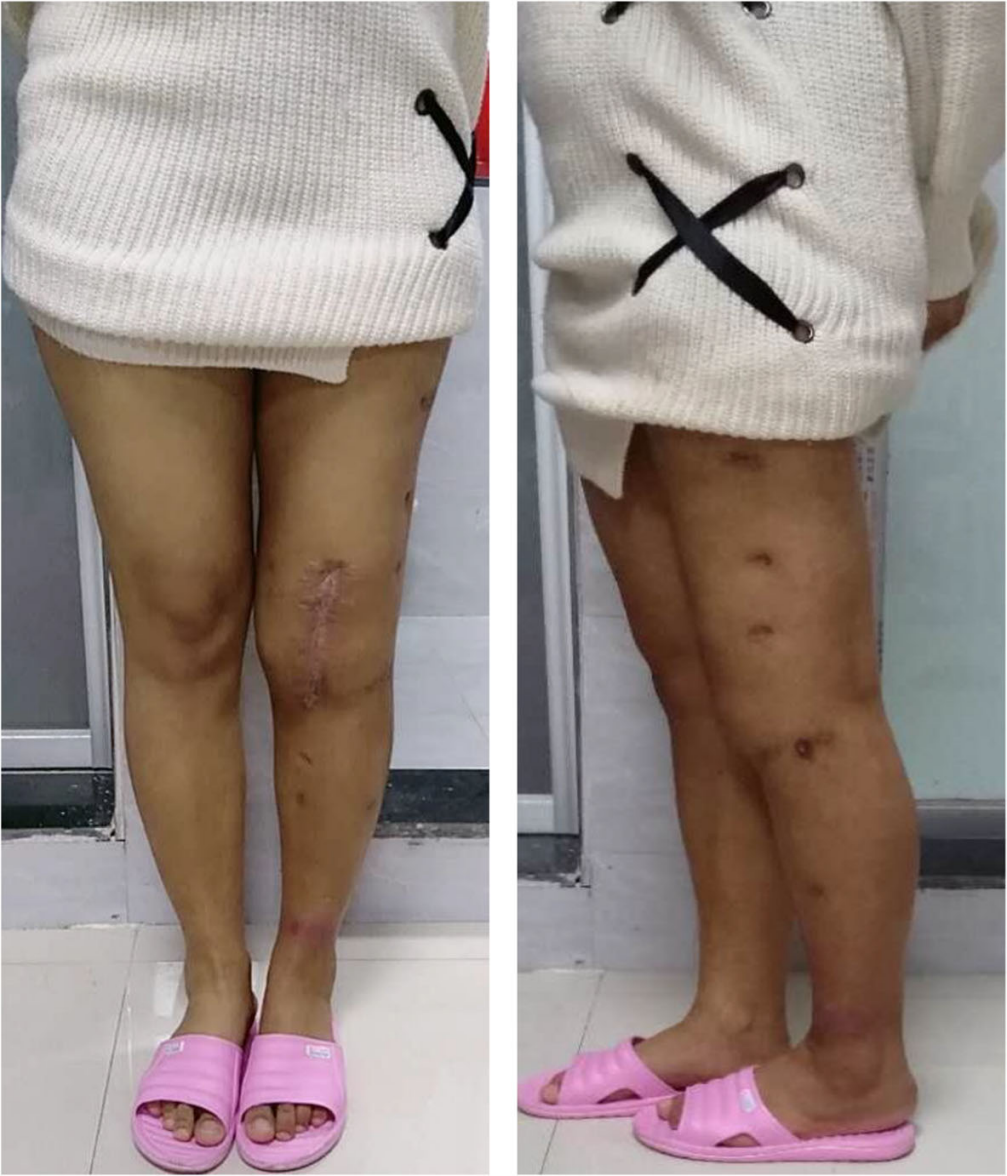
Good alignment at the anteroposterior and lateral positions (6 years postoperatively)

The ESR and CRP concentrations for all 24 patients returned to normal within 5.1 months (2–6 months) postoperatively. There was no reported recurrence of TB postoperatively. Radiographic images after removal of the external fixator revealed that the mean leg-length discrepancy was 2.7 cm (0.8–4.9 cm) after fusion, and the mean postoperative alignment was 8.7° flexion (5–15°) and 5.3° valgus (5–10°) (Figs. 6, 7 and 8). No patient had a significant rotational deformity. The mean Lysholm score improved significantly from 36.8 (28.0–49.0) preoperatively to 79.5 (65–86) at the final follow-up (*p* < 0.0001).

## Discussion

In the first-ever high-level meeting on TB held by the United Nations, sustainable development goals and the End TB Strategy of the World Health Organization were reaffirmed [1]. The strategy includes a 2020 milestone that no TB patients and their households will face high costs as a result of TB disease. However, because of the high incidence of TB in developing countries and underdeveloped areas, where patients with knee joint TB often do not seek medical attention until end-stage disease, the difficulty and cost of treatment are expected to be higher [21, 22]. Currently, none of the reported joint fusion techniques has been proven to be the best choice for the treatment of end-stage knee joint TB. Therefore, evaluating existing joint fusion techniques and developing new ones should be the top most priority, most often carried out by clinical orthopedic surgeons.

This study was conducted in southwest China, and most study participants are Tibetan. The local transportation options and economic situation are relatively poor, and there is much physical and weight-bearing labor in the life of these patients. Most are young or middle-aged, and arthrodesis can be used as a transitional operation to control inflammation. After inflammation is controlled, a second-stage knee replacement can be selected to restore joint function, whereas the first-stage knee arthroplasty has a higher cost and is accompanied by a higher risk of TB recurrence [23, 24]. In addition, for people with economic difficulties, especially for older patients, arthrodesis can also be used as the final treatment to relieve pain and improve quality of life. For these reasons, these authors hoped to adopt an appropriate arthrodesis technique as a treatment of end-stage TB of the knee with low cost, no need for secondary operation, allowance for immediate weight-bearing, and good curative effect. In this technique, thorough debridement of the knee joint was performed, followed by external fixation with the Ilizarov fixator. Anti-TB drugs were administered preoperatively, intraoperatively, and postoperatively.

The local use of anti-TB drugs after knee joint puncture has been recognized by researchers as having the advantages of being a simple operation, using low drug dosage, using a high local anti-TB drug concentration, and having obvious curative effect, among other benefits [25]. However, the local use of anti-TB drugs intraoperatively remains to be a controversial issue. Opposing scholars believe that the absorption of TB drugs into the blood will increase the patient’s drug resistance. However, in terms of total duration of use, anti-TB drugs cannot be regarded as antibiotics in the true sense, so these authors support the intraoperative local use of small doses of anti-TB drugs, specifically two doses of isoniazid, ethambutol, and streptomycin, all of which are fungicides. Before closing the incision, drugs were evenly placed into the joint cavity, and no adverse effects were found. The hypothesis is that local use of low-dose anti-TB drugs can have the advantage of high local drug concentration without poisoning. Of course, it is important to note that the amount and type of anti-TB drugs used in the operation must be confirmed by further research. As knee TB has spread to the whole joint during its end stage, the standard chemotherapy regimen of 12–18 months is preferred for patients who need surgical treatment [26]. Recent studies have found that the number of newly confirmed cases of bone and joint TB is still high because of the increases in population mobility and drug resistance [27]. Based on the large number of TB patients in western China and patients with an unknown medical history, clinical observation also shows a rebound trend in the number of drug-resistant TB patients. In addition, the transportation options and economic conditions for most of these patients remain to be limited; therefore, to prevent disease recurrence in treated patients, oral anti-TB drugs were recommended to be taken for 18 months. The current follow-up results show no cases of recurrence of TB.

The thorough debridement of TB arthritis is still the key to optimal curative effect, which can eradicate infected tissues to lower disease recurrence. All cartilage, dead bone, caseous tissue, inflammatory synovium, and abscess must be removed as far as possible. Preoperative CT can indicate the location and volume of dead bones. However, for some concealed abscesses and inflammatory soft tissues, especially in the end-stage of knee TB, there are mostly gravitation abscesses, which often spread to the thigh and calf [24]. Therefore, these authors recommend routine preoperative knee MRI. Thigh and calf MRI can also be performed when necessary to understand the scope and location of the abscess, which is helpful to achieve full removal of the abscess. Therefore, radiography, CT, and MRI examinations before the end-stage TB knee surgery are indispensable examinations.

Every arthrodesis technique for knee joint has its advantages and disadvantages. Internal fixations, including intramedullary nailing and plating, have seen a high rate of joint fusion and good stability of knee joint; however, extensive incision leads to long exposure time and more local soft tissue injury, which may trigger postoperative infection. Moreover, for patients with infectious diseases, whether the local internal fixation can be placed in first-stage surgery is still controversial. Patients with TB mostly have soft tissue sinus cavities, thin skin tissue, and other related conditions, and the placement of internal fixation can increase the risk of wound nonunion [28, 29]. Methods that use cannulated screws with or without external fixator have a low risk of postoperative infection, and the operation procedure is considered simple [24, 30]. However, joint stability is relatively poor. Triangular or some circular external fixators that do not pass through the articular cavity often provide good stability and have convenience of removal, but the rate of joint fusion is low and the fusion time is long [31, 32]. In addition, there may be pin tract infection and inconvenience in dressing the wound. Combined with the characteristics of end-stage knee TB and the local poor conditions of transportation, economy, labor, and other factors for most patients, the authors of this study recommend the Ilizarov technique for knee arthrodesis. Since its invention, the Ilizarov external fixator has been used in orthopedic fixation of the whole skeletal system, and the effect is satisfactory. In the past, these authors have mainly used this technique for lower limb orthopedics and bone transporting operations. This technique has the following advantages: (1) it can be pressurized at 360°, fixed accurately, and allows immediate weight-bearing, which cannot be achieved by any kind of internal and other external fixation; (2) if the fusion angle and pressure are not ideal in the early postoperative stage, it can be adjusted; (3) its installment is far from the affected site, which can minimize the dissemination of TB and provide better conditions for wound care; (4) this approach of external versus internal fixation is more appropriate for patients with sinus cavities, skin defects, thin skin tissue, or a large number of bone defects; (5) this apparatus can be inserted after releasing the tourniquet and closing the incision, leading to a reduced duration of surgical and tourniquet exposure; (6) the frequency and time of intraoperative radiographic fluoroscopy during the procedure of it are even less compared to those of internal fixations; (7) the rings of this apparatus used for this study were made of steel rather than carbon, so the material price of it was less expensive compared to that of internal fixations; and (8) it is convenient to remove the fixator after recovery, which can be accomplished under local anesthesia in the local hospital [33–35]. However, this technique also has some disadvantages, such as the heavy weight of the fixator, inconvenience in dressing and nursing, and a high risk for pin tract infection [36, 37]. For some extremely obese patients, the selection of the ring for the thigh is also a concern because of the large ring size required, so it is necessary to fully communicate with the patients and their families for better psychological preparation before surgery. In addition, due to the difficulty of the operation using Ilizarov external fixator, this technique requires extensive experience and high skills, which is also a challenge for orthopedic surgeons.

Pin tract infection after use of Ilizarov technique is still considered a common complication, which may be related to skin tension and pin temperature [36]. In the process of pin piercing, close attention should be paid to the change of tension. If the tension is high, partial release should be made around the pin tract. The authors of this study have achieved this release by cutting a 2-mm opening in the tissue along the limb in the distal part of the pin. Then, the pin was wrapped with alcohol-soaked gauze while penetrating through the proximal tissue, and bone hammer was used in penetrating through the contralateral tissue, which can reduce the overheating of the Kirschner wire during the drilling process, thus minimizing thermal burns to the skin and soft tissue. Around the fixed pin, gauze was used to squeeze and fix the skin to reduce the friction between the skin and the pin and to minimize the possibility of infection. After surgery, the pin tract was washed with saline, and mupirocin ointment was applied to the pin tract to avoid severe pain during dressing changes. Satisfactory results were obtained in the prevention of infection as evident in the follow-up data. Only two patients had mild infection in one pin tract, which was relieved after rest and intensive pin tract care.

In this study, 95.8% of the cases of primary bone union were achieved during the follow-up, and only one patient developed delayed union without a secondary operation. The 95% confidence intervals for primary bone fusion rate is (0.872–1). The mean time to primary bone union was 6.4 months, and the complication rate was recorded to be at 16.7%. The 95% confidence intervals for complication rate is (0.006–0.327). The results of the treatment are in agreement with those of the previous studies, which used various arthrodesis techniques for infected knees or total knee arthroplasty failure (mean fusion rate, 64–100%; mean fusion time, 4.5–12.4 months; complication rate, 43–100%) [38–42]. The inflammation index including white blood cell count, ESR, and CRP returned to normal after an average of 5.1 months postoperatively, which is consistent with previous reports [43]. The mean Lysholm score at the final follow-up (79.5) improved significantly compared with that before surgery (36.8), which suggests that the patients had better function of knee joint and higher quality of life after joint fusion [44].

The angle of knee joint after fusion has always been a controversial issue. To reduce the further shortening of the limbs, most experts advocate knee joint fusion in extension. When the pelvis is raised excessively at the 0° flexion of the knee, the patients compensate for the stiffness of the knee joint by abduction of the hip joint and outward circle of the lower limb, which will increase the energy consumption by 25% during walking. Knee fusion with the flexion of 10°–15° can provide a better sitting position and improve gait; however, an angle of about 15° will increase the shortening by about 2 cm [45]. It is also advocated that the flexion of 5°–10° can better solve the contradiction between energy consumption and limb shortening [46]. In this study, the flexion of 5°–10° was performed for all patients during the operation. The average flexion after fusion at the final follow-up was 8.7°, and the average limb shortening was 2.7 mm. Nonetheless, the gait of all patients was good, and most of the patients were satisfied with the treatment effect. These authors speculate that the reason for the limb shortening may be osteoporosis and bone compression after long-term external compression fixation; therefore, whether the treated patients need bone grafting and calcium supplement is still an issue that needs to be addressed.

The results of this study have shown that that the Ilizarov technique is an excellent choice for the treatment of end-stage knee TB; however, this technique has some disadvantages. Limitations of this study are that it is retrospective and the sample size is relatively small. Therefore, a prospective randomized controlled trial with a large sample size is needed to further confirm the curative efficiency of this technique.

## Conclusion

The Ilizarov technique has been considered as an ideal method for knee joint arthrodesis in treating end-stage TB of the knee. It has several advantages, including low cost, not needing secondary operation, allowance for immediate weight-bearing, a high rate of fusion, and minimal complications. This study can provide a reference to support physicians and patients in need of this treatment; however, further extensive studies are needed to comprehensively evaluate its curative effect.

## Data Availability

Data and material related to this study is available from the corresponding author on reasonable request.

## Abbreviations

TB: Tuberculosis
ESR: Erythrocyte sedimentation rate
CRP: C-reactive protein
CT: Computed tomography
MRI: Magnetic resonance imaging
